# Analysis of drug sensitivity of human high-grade osteosarcoma in a chick chorioallantoic membrane (CAM) model: a proof of principle study

**DOI:** 10.1186/s13104-020-05269-x

**Published:** 2020-09-15

**Authors:** Wiebke K. Guder, Wolfgang Hartmann, Marcel Trautmann, Jendrik Hardes, Eva Wardelmann, Maurice Balke, Arne Streitbürger

**Affiliations:** 1 Department of General and Tumor Orthopedics, University Hospital Muenster, Albert-Schweitzer-Campus 1, Building A1, 48149 Münster, Germany; 2 Division of Musculoskeletal Oncology, University Hospital Essen, Hufelandstrasse 55, 45147 Essen, Germany; 3 Gerhard Domagk Institute of Pathology, University Hospital Muenster, Albert-Schweitzer-Campus 1, Building D17, 48149 Münster, Germany; 4 Sportsclinic Cologne, Ostmerheimer Strasse 200, 51109 Cologne, Germany

**Keywords:** Chorioallantoic membrane model, CAM, Osteosarcoma, Primary cell culture

## Abstract

**Objective:**

Multi-agent chemotherapy is an important pillar in treatment of high-grade osteosarcoma. In an effort to improve patient survival, it is imperative to determine the effectiveness of new substances. The objective of this study was to investigate whether the chick chorioallantoic membrane (CAM) model can be used to analyze drug sensitivity in high-grade osteosarcoma.

**Results:**

Spare biopsy tissue from five patients diagnosed with high-grade osteosarcoma was transferred into non-immortalized primary cell culture. After a pre-incubation period of 10 days, fertilized chick eggs were inoculated with primary tumor cells suspended in extracellular matrix gel. On day 16, treatment with 20 µmol/l doxorubicin (n = 4) or 25 µl of culture medium (n = 6) was performed for 24 h. CAM membranes were documented macroscopically, harvested and examined histologically. Transfer of biopsy specimens into primary cell culture was successful in all cases. 50% (n = 10) of eggs died after inoculation with tumor cells and before application of doxorubicin. No deaths occurred after application of doxorubicin. Histological examination found a response to doxorubicin in all four specimens. Based upon these results, the CAM model represents a promising preclinical alternative to animal experiments to determine drug sensitivity of osteosarcoma cells. Further research with regard to other substances and dosages appear justified.

## Background

High-grade osteosarcoma is a rare disease, mostly affecting children, adolescents and young adults. A combination of chemotherapy and wide tumor resection are the main pillars for successful treatment of these patients. The significance of chemotherapy becomes clear when considering how overall survival rates improved from 20% to more than 60% with the introduction of multi-agent chemotherapy [1]. Standard chemotherapeutic agents include doxorubicin, cisplatin, ifosfamide and methotrexate [2]. Patients are usually enrolled in multimodal clinical trials in an effort to improve treatment outcomes by comparing new approaches to a standard regimen. Current protocols intend patients to undergo neoadjuvant chemotherapy to evaluate response to chemotherapy as the percentage of tumor regression found in the resection specimen of the primary tumor. As a result, good and poor responders are assigned to different adjuvant chemotherapy groups, which vary with regard to duration and composition of remaining treatments [2]. However, evidence regarding the efficacy of this risk-stratified treatment approach is still lacking [3, 4]. Therefore, in case of poor response to chemotherapy, an antedated operation or modulation of administered chemotherapy might be desirable. However, no significant developments have been made with regard to new drugs in osteosarcoma protocols over the last few decades which may also be due to the lack of suitable preclinical individualized models having the power to reliably predict response [1, 3, 4].

Preclinical research currently relies on in vitro and mouse models for drug screens to identify promising substances that might be translated into a clinical trial [5]. The latter in vivo xenograft models tend to be lengthy and unable to yield short-term information that might be able to influence individual treatment approaches. Beyond that, there has been a paradigm shift in our scientific society: ethical recommendations challenge scientists to develop models that do not rely on animal experiments whenever possible. In this context, the chorioallantoic membrane (CAM) model is particularly interesting as the chick embryo is not considered to be a living animal until day 17 of development in most countries and therefore does not fall under animal experiment [6, 7]. Though successful engraftment of musculoskeletal tumors including human osteosarcoma cell lines has been reported and though there is some preliminary evidence that it may serve as a preclinical screening assay predictive for disease outcome, it is not in common use yet [8, 9].

Therefore, it is the objective of this study to analyze whether the CAM model may serve as a reliable preclinical drug-screening assay and if so, may even be predictive of response of the tumor to chemotherapy seen in the resected specimen of the primary tumor in a follow-up study.

## Main text

### Material and methods

#### Biopsy aliquots

Biopsy aliquots used in this study were obtained from five patients who underwent diagnostic incisional biopsy for suspected high-grade osteosarcoma at our clinic. Pathologists separated a biopsy aliquot from the existing tissue provided that sufficient tumor tissue was available without impairing standard diagnostic procedures. Histological examination confirmed high-grade osteosarcoma in all five cases.

#### Aliquot preparation

Biopsy aliquots were minced in a petri dish using a scalpel under laminar airflow. 1–2 ml DMEM was added and the biopsy tissue strained through a sieve (Corning^®^ Costar^®^ Cell Strainer 100 µm). The suspension was centrifuged for 3 min at 800 revolutions per minute (RPM). Then, the supernatant was pipetted into a T75 tissue culture flask. The remaining pellet was digested for 15 min at 5% CO_2_ and 37° Celsius using 5–7 ml trypsin (trypsin EDTA, 0.25%). Tissue digestion was inhibited adding 5–7 ml DMEM, and the suspension was centrifuged again as mentioned above. The supernatant was removed, the pellet solved in DMEM and then transferred into a T25 tissue culture flask.

#### Primary cell culture

All primary cell lines were grown in DMEM (Gibco™ Dulbecco’s Modified Eagle Medium, high glucose, GlutaMAX™) supplemented with 10% fetal bovine serum (FBS), 1% l-glutamine and 1% penicillin/streptomycin on T25, T75 or T175 tissue culture flasks in a humidified atmosphere of 5% CO_2_ at 37° Celsius. Tissue culture flasks were coated in poly-l-lysine and washed with DPBS (Gibco™ Dulbecco’s phosphate-buffered saline) in preparation of tumor-derived single cell suspensions. During incubation, vitality of primary cell cultures was checked on a daily basis under a light microscope. Cell culture medium was exchanged and the cell lines expanded depending on cell adherence, cell count and quality of culture medium.

#### Chorioallantoic membrane (CAM) model

CAM protocols as proposed by Sys et al. and Balke et al. were adapted to our study design [10, 11]. Fertilized, pathogen-free eggs (n = 88) were purchased from and delivered by VALO BioMedia GmbH (Osterholz-Scharmbeck, Germany). These were kept in a wine cooler at 12–14° Celsius and a humidity of 70–80% as shortly as possible. After transfer into the incubator, egg development was counted in days and eggs were exclusively handled under laminar airflow when outside the incubator. Incubation was performed in a sideways position at 37.5°–38° Celsius and a humidity of 70–80%. On day 3 of egg development, a 1–2 mm hole was poked into the shallow pole of the egg, and 3 ml of egg white was withdrawn from the egg to separate the CAM membrane from the eggshell. The hole was then sealed using adhesive tape. Subsequently, a circular window of approximately 2 cm in diameter was cut into the eggshell. The egg white was then returned into the egg and the circular window was sealed by adhesive tape. Eggs without an embryo were removed from the experiment. Egg vitality was checked on a daily basis and devitalized eggs were removed from the incubator. On day 10 of egg development, cells were harvested from primary cell culture and suspended at 10^6^ cells/100 µl in extracellular matrix gel (ECM). Per egg (n = 20), 100 µl of this suspension were topically applied in layers on the CAM membrane after careful superficial incision performed with a scalpel to facilitate ingrowth of the tumor on the CAM. Daily check-ups of egg vitality and removal of dead eggs was continued. On day 16 of egg development, remaining eggs (n = 10) were randomly divided into a treatment and a control group and either topically applied with 25 µl of 20 µmol/l doxorubicin (n = 4) or 25 µl cell culture medium (n = 6). After 24 h, on day 17 of egg development, the CAMs were covered in formaldehyde solution for 30 min before the area of interest (macroscopic tumor growth) was excised and processed for histological examination.

#### Histological analysis

Tissue workup followed standard routines including fixation of the tumors in 3.7% buffered formalin for 24 h, standard dehydration and paraffin embedding followed by the preparation of paraffin blocks. Hematoxylin and eosin (H&E) and Verhoeff’s van Gieson (EVG) staining were performed on 3 µM tissue sections following standard protocols.

### Results

#### Primary cell culture

Transfer of processed biopsy aliquots into non-immortalized primary cell cultures including in vitro expansion of these tumor-derived single cell suspensions was successful in all specimens (n = 5).

#### Chorioallantoic membrane (CAM) model

Overall, egg survival was 86.3% (n = 76/88) at day 3, 54.5% (n = 48/88) at day 10 and 37.5% (n = 33/88) on day 17 of egg development. 50% of eggs (n = 10) inoculated with tumor cells on day 10 (n = 20) died before application of doxorubicin on day 16. All remaining eggs (n = 10) survived until termination of the experiment 24 h after application of doxorubicin.

#### Histological analysis

**Fig. 1:**
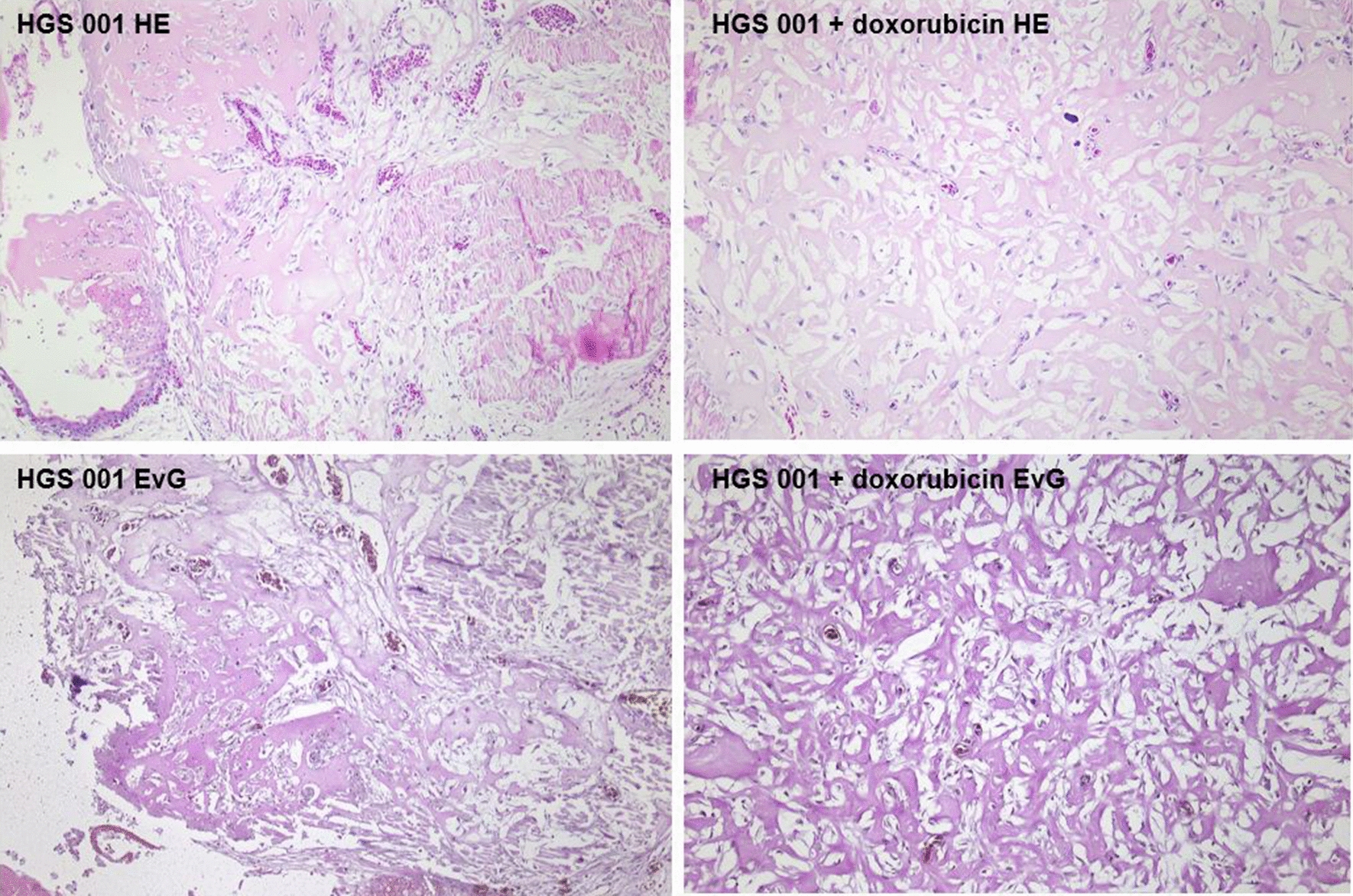
HE and EVG staining of tumors grown from the same primary cell culture (HGS001) without (left side) and after (right side) application of doxorubicin

**Fig. 2:**
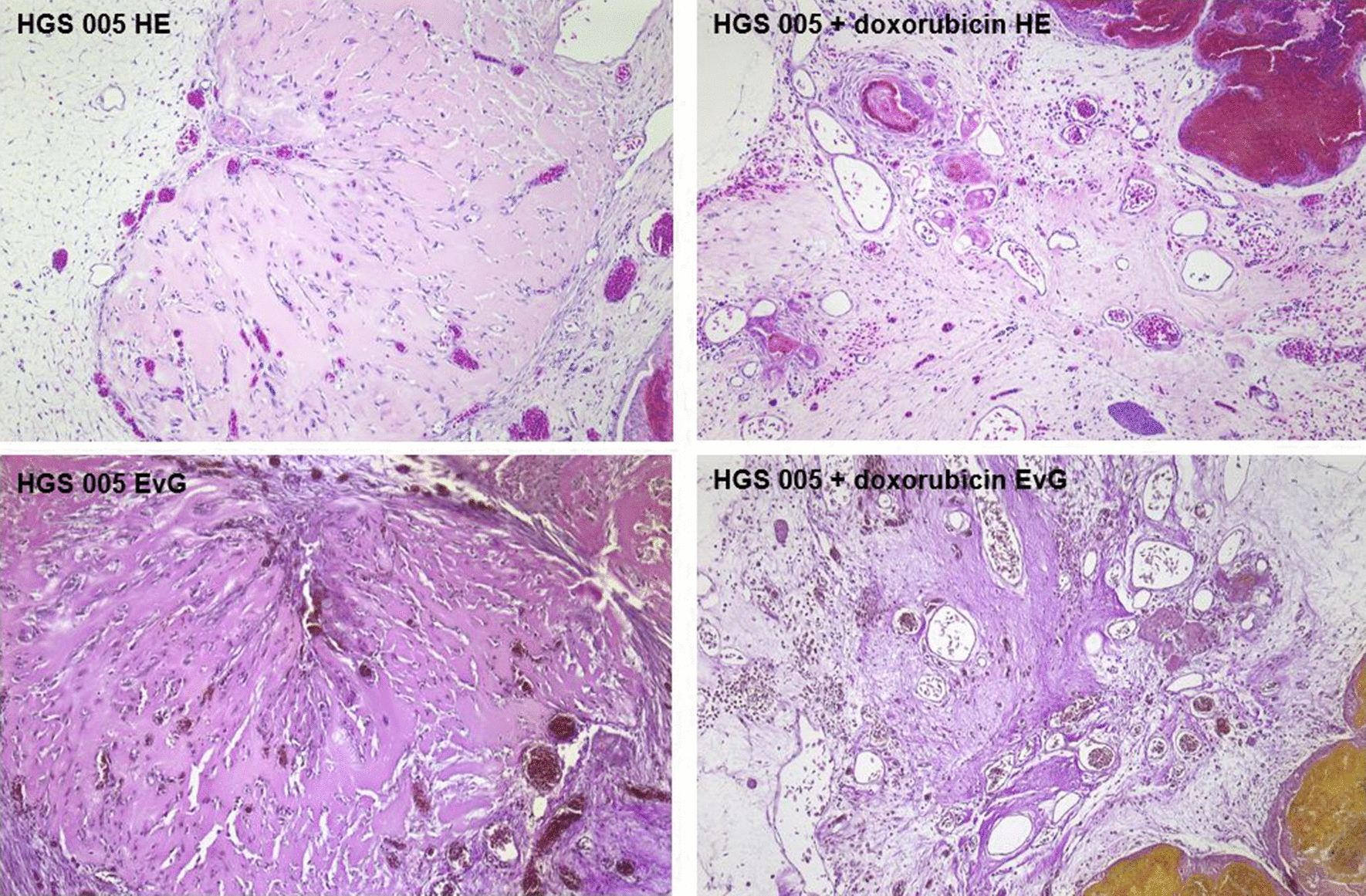
HE and EVG staining of tumors grown from the same primary cell culture (HGS005) without (left side) and after (right side) application of doxorubicin

Viable, CAM infiltrating tumors recapitulating osteosarcoma tumors with extensive osteoid formation were found in all specimen gained from the control and treatment group with the latter showing partial necrosis and hemorrhage as correlates of tumor regression while osteoid formation was retained (Figs. 1 and 2).

### Discussion

In this preliminary study investigating the aptitude of the CAM model in testing drug sensitivity of high-grade osteosarcoma, we were able to show a therapeutic effect of doxorubicin on high-grade osteosarcoma grown on the CAM from tumor-derived single cell suspensions after prior expansion in non-immortalized primary cell culture.

While Sys et al. and others have reported the CAM to be a suitable model to grow musculoskeletal tumors and have suggested it as a model to test drug sensitivity, to our knowledge this study is the first one to prove a therapeutic effect of a chemotherapeutic agent on a tumor grown from primary osteosarcoma cells in the CAM [8, 9]. The results presented in our study, such as a high rate of embryo deaths throughout the incubation process, are in accordance with other reports in literature [8, 9] with Sys et al. reporting a rate of 20% of embryo deaths during the incubation period in their 2012 study [8]. Despite the fact that roughly two-thirds of the egg population died in the process of incubation and after inoculation with tumor cells, we still believe the CAM model to be a prospective alternative to other established mammalian models. It is a short-period, low-cost model from which conclusions can still be drawn from an adequate number of surviving eggs by repetition of the same protocol if need be. Further interest in this model may be drawn from the fact that it is not considered an animal experiment and therefore is in accordance with endeavors to avoid the employment of animals in preclinical research. The only alternative to date, which might manage without animal experiments altogether are bioengineered systems trying to mimic key aspects of human tumor microenvironments. Studies, such as an investigation by Marturano-Kruik et al. examining the biomechanical regulation of drug sensitivity in an engineered model of human tumor need to be compared with the results gained in CAM experiments to determine each model’s aptitude in the future [12].

Therefore, follow-up studies are necessary to assess the significance this proof of principle has on future research projects. Particularly, the representation and comparability of tumor cell clones expanded in cell culture compared to the entire tumor needs to be verified. Also, other chemotherapeutic agents and their impact on tumor growth on the CAM need to be evaluated. Dose dependency of substance response for each substance has to be analyzed and compared with or translated into a clinical setting. Furthermore, additive effects caused by a combination of chemotherapeutic agents will have to be observed and compared with the CAM, other models and clinical experiences. Assessment on whether response to chemotherapy can be classified by grade of tumor necrosis and whether results compare with clinical results are necessary as tumor specimens grown on the CAM are small compared to the primary tumors they are supposed to represent. And lastly, it remains to be seen whether the human and CAM environment are similar enough to yield the same results. For example, the egg embryo’s delayed development of an immune system [6] might affect tumor growth and response to chemotherapy and produce results that might be different in a human.

## Conclusion

We believe the results presented in this study prove that analysis of drug sensitivity is possible on the CAM and follow-up studies investigating this model’s clinical applicability are justified.

## Limitations

Due to a very small number of evaluated eggs (n = 10) in this preliminary proof of principle study, investigation of tumor volumes with and without application of a chemotherapeutic agent did not yield meaningful results yet and further assessment will be necessary in larger follow-up studies.

## Data Availability

The datasets used and/or analyzed during the current study are available from the corresponding author on reasonable request.

## Abbreviations

CAM: Chorio-allantoic membrane
DMEM: Dulbecco’s Modified Eagle Medium
DPBS: Dulbecco’s phosphate-buffered saline
ECM: Extracellular matrix
FBS: Fetal bovine serum
RPM: Revolutions per minute
